# Ameloblastic Fibroma of the Mandible Presenting as a Dentinoid Formation in an 8‐Year‐Old Child: A Case Report

**DOI:** 10.1002/ccr3.70169

**Published:** 2025-01-31

**Authors:** Priyambada Karna, Varun Rastogi, Dilasha Dhungel, Sandhya Chaurasia

**Affiliations:** ^1^ Department of Oral & Maxillofacial Pathology Universal College of Medical Sciences Bhairahawa Nepal

**Keywords:** ameloblastic fibroma, benign, dentinoid, malignant transformation, odontogenic tumor

## Abstract

Ameloblastic fibroma is an uncommon, benign, mixed odontogenic tumor characterized by the presence of both epithelial and mesenchymal components, without the formation of hard tissue. It constitutes approximately 1.5%–4.5% of all odontogenic tumors and predominantly affects the posterior mandible. Most cases are observed in individuals under 22 years of age, with a slight predilection for males. The initial presentation typically involves a painless, slow‐growing mass, often discovered incidentally during routine radiographic examinations. This lesion carries a risk of recurrence and potential for malignant transformation. We present a case of an 8‐year‐old female child who presented with a painless swelling in the right posterior mandibular region for 6 months, which was histologically characterized by dentinoid formation.

**Summary**

- Ameloblastic fibroma is a rare, benign, mixed odontogenic tumor primarily affecting children and adolescents.
- Early detection through clinical and radiographic evaluation is essential to prevent significant jaw deformities.
- Surgical enucleation or conservative excision remains the treatment of choice, with regular follow‐up to monitor for recurrence.

## 1 Introduction

Ameloblastic fibroma (AF) is a rare, benign odontogenic tumor, accounting for approximately 1.5%–4.5% of all odontogenic tumors. It was first described by Krause in 1891, but it was later recognized as a distinct entity by Thoma and Goldman in 1946 [1]. It is more commonly seen in children and adolescents, especially within the first two decades of life. There is a slight predilection for males [2], and the posterior mandible is the most frequent site of involvement. It is often associated with impacted or unerupted teeth [3].

Clinically, AF typically presents as a slow‐growing, painless swelling, which may remain undetected until incidental discovery during routine radiographic examination. Radiographically, it often appears as a well‐defined, unilocular or multilocular radiolucent lesion often with sclerotic radiopaque borders [1]. Although considered benign, AF has the potential for recurrence and, in rare instances, malignant transformation into ameloblastic fibrosarcoma, emphasizing the importance of early diagnosis and appropriate management.

Histopathologically, AF consists of both epithelial and connective tissue components. The epithelial component shows proliferating islands, cords, and strands of odontogenic epithelium with a peripheral layer of cuboidal or columnar cells and a central area resembling the stellate reticulum. The connective tissue component, resembling dental papilla, contains spindle, and angular cells within a myxomatous stroma of delicate collagen [2, 3]. Treatment options typically involve enucleation and curettage, surgical excision, partial resection, and reconstruction. This case report discusses a rare presentation of AF in an 8‐year‐old female child, who presented with a painless swelling in the right posterior region of the mandible, which was histologically characterized by dentinoid formation.

## 2 Case History/Examination

**FIGURE 1:**
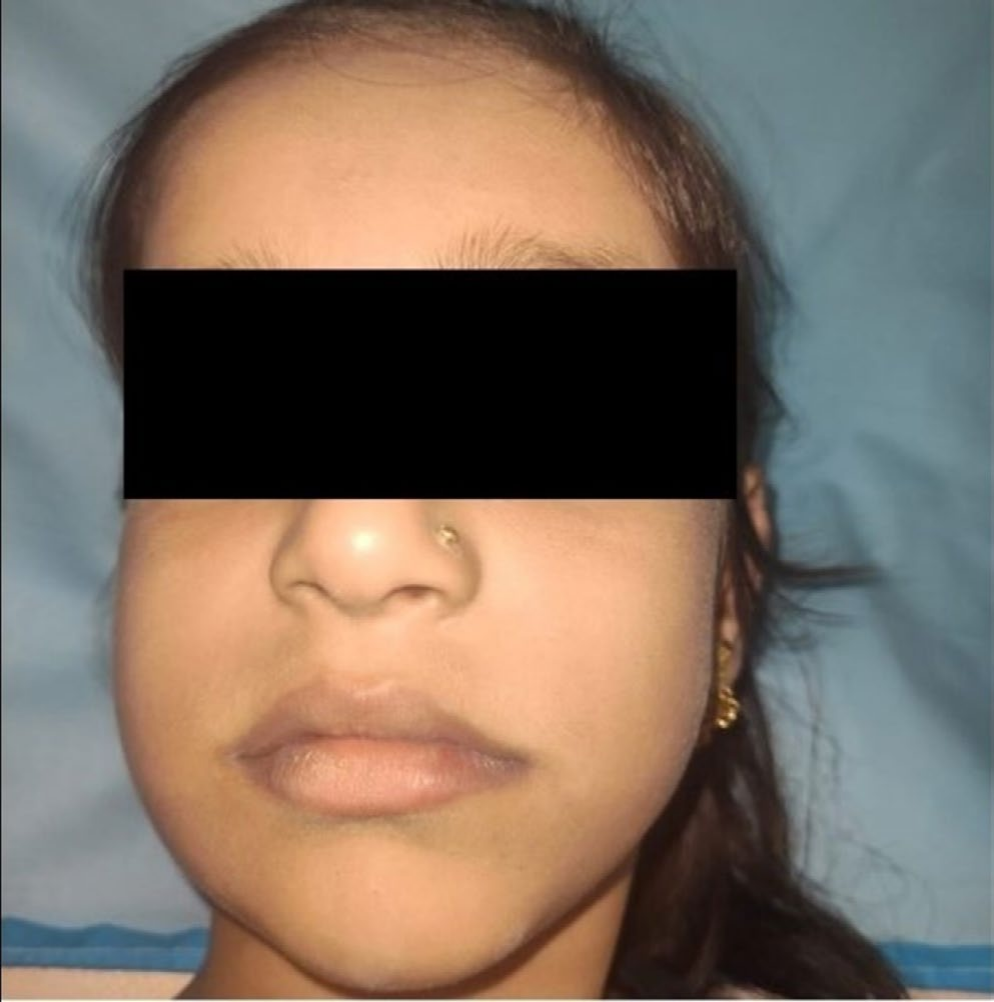
Extraoral presentation.

An 8‐year‐old female child presented to the Department of Oral Medicine and Radiology with a swelling in the right posterior mandibular region, measuring approximately 4 × 3 cm, involving the body and angle of the mandible, persisting for the past 6 months (Figure 1). The swelling was firm, nontender, and exhibited buccal and lingual cortical bone expansion on examination.

## 3 Methods

**FIGURE 2:**
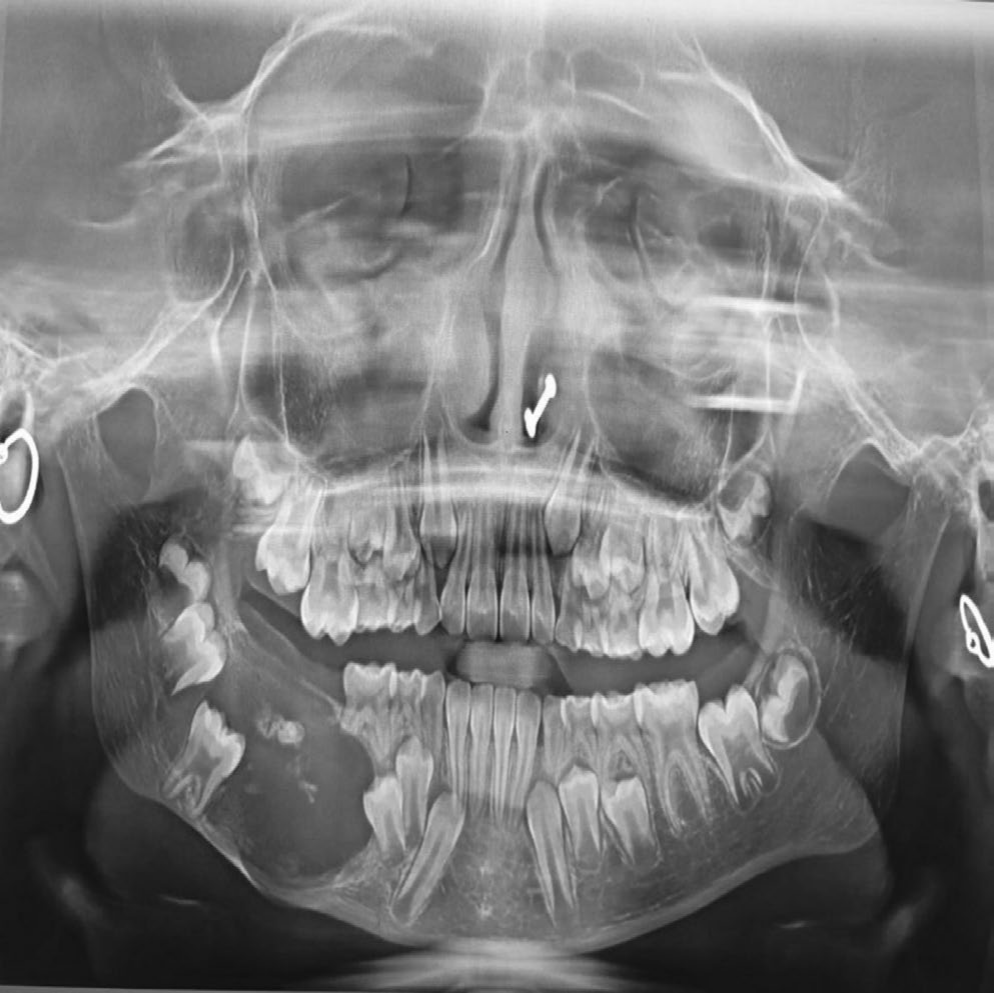
Orthopantomography reveals radiolucency involving posterior region of right side of mandible.

Aspiration yielded no fluid. The orthopantomogram showed a radiolucent lesion extending from the region of tooth 54 to the mandibular ramus, with areas of bony spicules. Erupting teeth 46, 47, and 48 were noted, along with resorption of teeth 84 and 85 (Figure 2).

**FIGURE 3:**
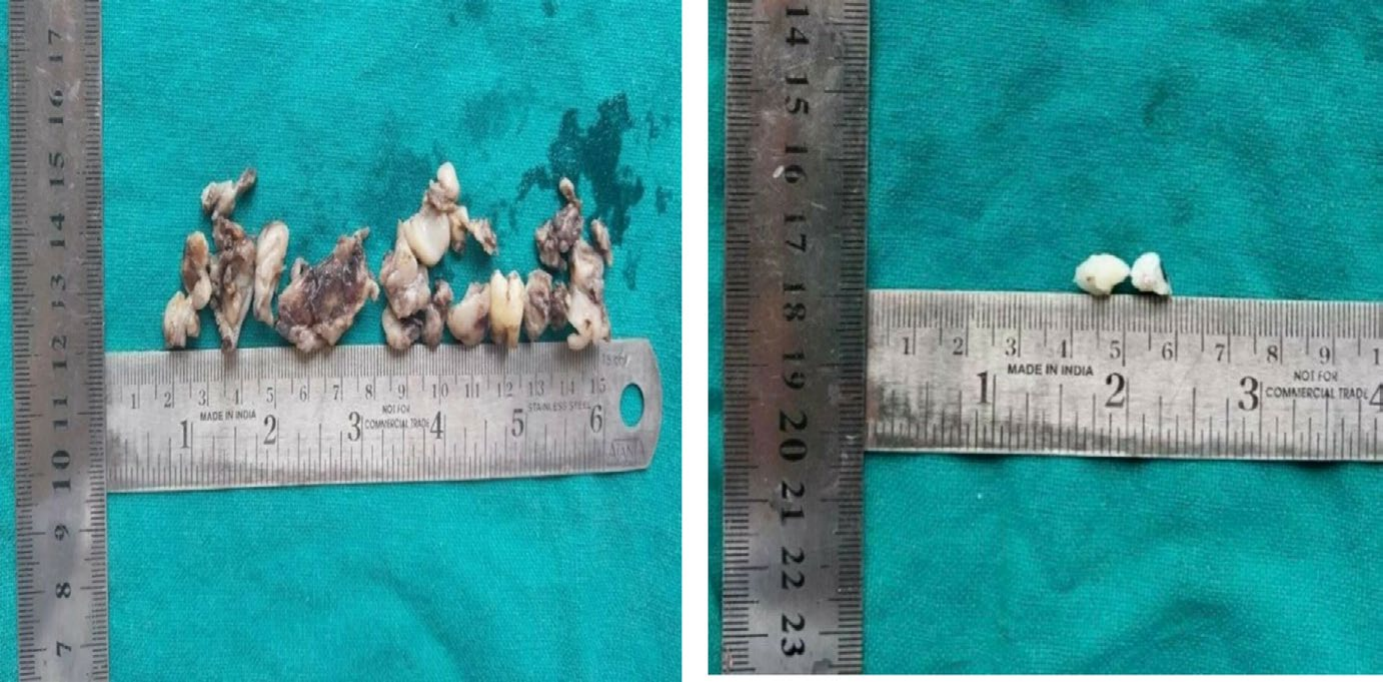
Macroscopic findings.

**FIGURE 4:**
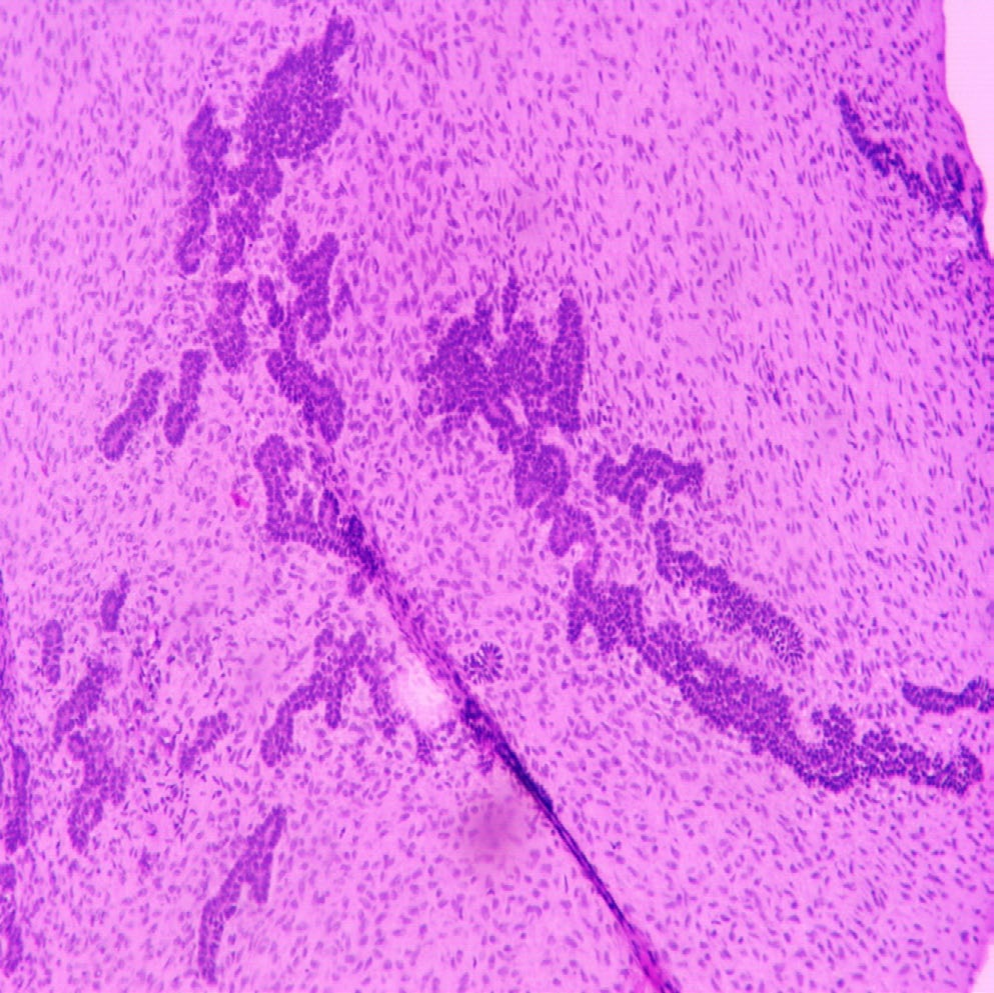
Low power view (4×) showing odontogenic epithelium.

**FIGURE 5:**
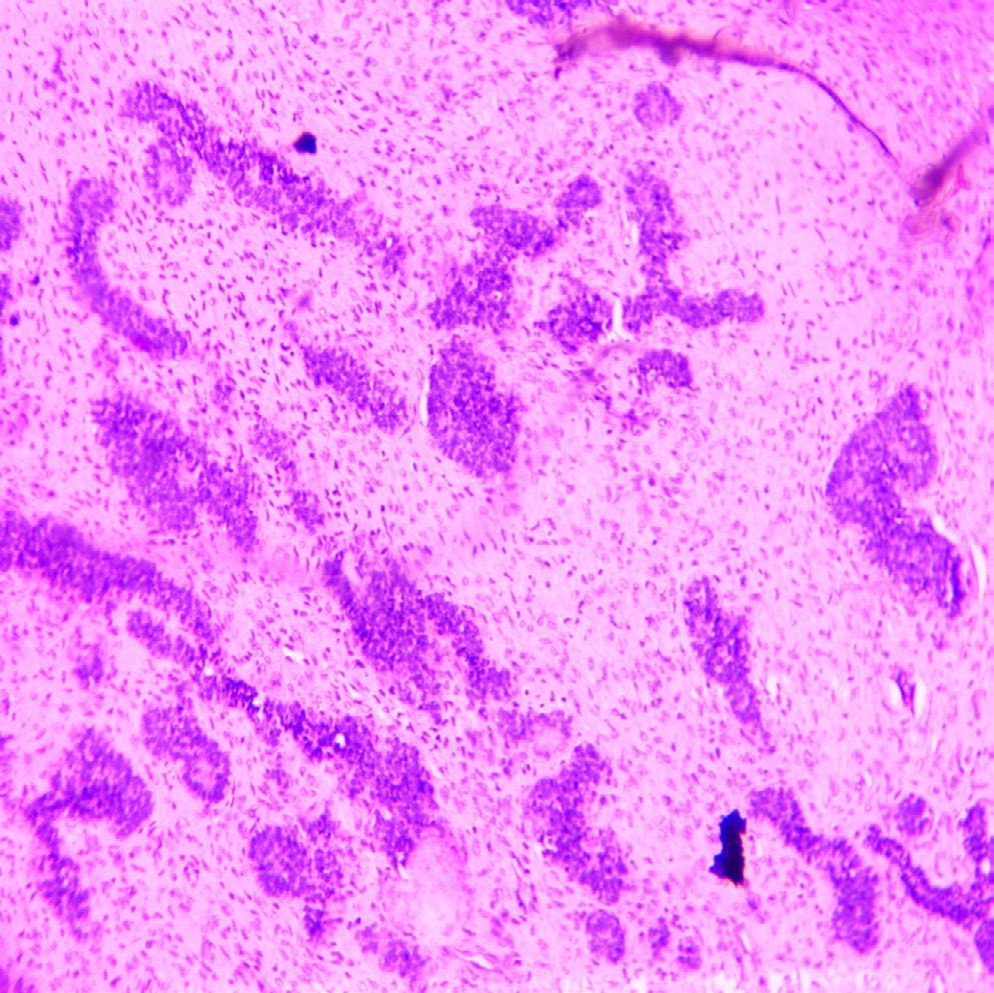
Lower magnification (10×) showing islands, nests, strands, and cords of odontogenic epithelium.

**FIGURE 6:**
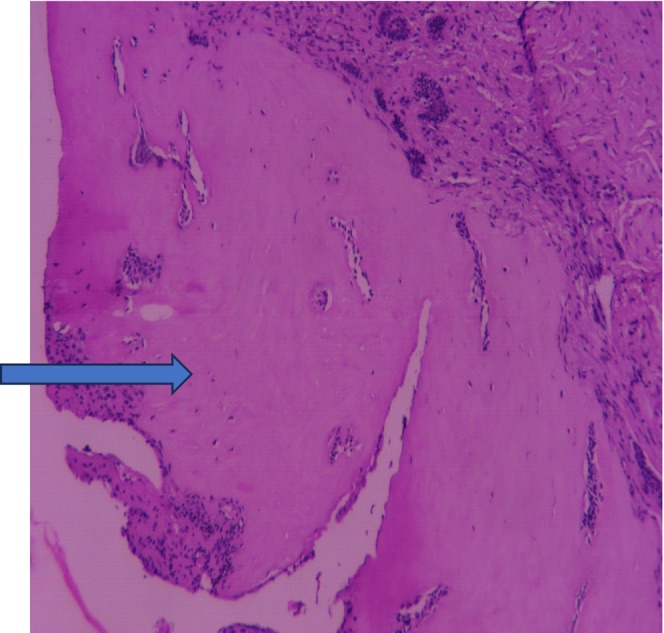
Lower magnification (10×) showing homogenous eosinophilic area resembling dentinoid.

The differential diagnoses considered were juvenile ossifying fibroma, central ossifying fibroma, and odontogenic myxoma. An incisional biopsy was performed under local anesthesia, and the specimen was preserved in a 10% formalin solution for further histopathological analysis. Macroscopic examination revealed two bits of tissues, exhibiting white to dark brown in color. The consistency was firm to hard and measured approximately 1.9 × 0.6 cm (Figure 3). Histopathological examination by routine hematoxylin (H and E) staining showed odontogenic epithelium and ectomesenchyme (Figure 4). Lower magnification examination showed islands, nests, strands, and cords of odontogenic epithelium within ectomesenchyme (Figure 5). Odontogenic epithelial islands exhibited peripheral tall columnar cells with hyperchromatic, round nuclei, and reversed polarity, along with central stellate cells in some areas. The ectomesenchyme was highly cellular, containing round‐, oval‐, spindle‐shaped, and angular cells resembling dental papilla. Large homogeneous eosinophilic areas resembling dentinoid (Figure 6) with entrapped odontogenic epithelial cells, moderate vascularity, and the areas of hemorrhages were also seen. The overlying epithelium was parakeratinized stratified squamous epithelium. Based on the correlation of clinical and histopathological results, a diagnosis of AF with dentinoid was confirmed for the lesion.

## 4 Conclusion and Results

After complete enucleation followed by curettage, the patient was closely monitored during follow‐up visits. 6 months postoperatively, there were no signs of disease recurrence.

## 5 Discussion

AF is a rare, benign, mixed odontogenic tumor, classified by the World Health Organization (WHO) as a lesion containing both epithelial and ectomesenchymal components, without the formation of hard tissue. AF most commonly occurs during the second decade of life and is observed three times more frequently in the mandible than in the maxilla. It is often associated with deciduous molars or the permanent first molar, with 80% of cases involving these teeth. Furthermore, 75% of cases are linked to impacted teeth [1, 2]. Clinically, AF presents as a slow‐growing, painless swelling that can lead to jaw expansion, displacement of erupted teeth, and, in rare instances, facial disfigurement. Most cases remain undiagnosed until incidentally discovered during routine radiographic evaluations [4, 5].

The pathogenesis of AF has been explored extensively, and various theories have been proposed. Cahn and Blum's maturation theory suggests that AF progresses through continuous differentiation into ameloblastic fibro‐odontoma (AFO) and subsequently into odontoma, which is considered a hamartoma [6]. However, this theory lacks wide acceptance for two primary reasons: residual or recurrent AF does not show evidence of developing hard tissue tumors at advanced stages, and AF typically manifests after the completion of odontogenesis [7]. Reflecting these findings, the most recent WHO classification excludes AFO as a distinct entity, considering it an intermediate developmental stage of a tumor evolving from ameloblastoma to odontoma [5].

Ameloblastic fibrodentinoma (AFD), another rare mixed odontogenic tumor, shares histological features with AF, such as the presence of odontogenic epithelial nests or cords lined by tall ameloblast‐like cells, central stellate reticulum‐like cells, and ectomesenchymal tissue. However, AFD is characterized by the additional presence of tubular or dysplastic dentin. Both AFD and AFO harbor the BRAF p.V600E mutation, distinguishing them from odontomas, which lack this mutation. This genetic overlap, also observed in AF, raises questions about whether AFD and AFO represent separate entities, intermediate lesions leading to odontoma formation, or a combination of developing odontomas and AFs [8, 9].

Recent advancements in understanding the pathogenesis of AF highlight the roles of genetic, molecular, and epigenetic factors. Mutations in genes such as MSX1, PAX9, and BRAF p.V600E, along with alterations in Wnt/β‐catenin signaling pathways, have been implicated in disrupting odontogenesis‐related processes. Dysregulated signaling pathways, including Notch, Hedgehog, and Fibroblast Growth Factor (FGF) pathways, also contribute to abnormal cell differentiation and proliferation in AF. Furthermore, disturbances in epithelial–mesenchymal interactions and epigenetic modifications, such as DNA methylation and histone changes, play a crucial role in tumor development [10].

Radiographically, AF typically presents as a unilocular or multilocular radiolucent lesion, often with sclerotic radiopaque borders. This appearance may mimic other odontogenic lesions, such as dentigerous cysts. Microscopically, AF consists of strands and islands of odontogenic epithelium resembling the early enamel organ and a cellular mesenchymal component resembling dental papilla. In recurrent cases, dentin formation, with or without enamel structures, may be observed. Immunohistochemically, the odontogenic epithelium shows positive staining for cytokeratin, the mesenchymal tissue for tenascin, and the basement membrane for vimentin. These findings help distinguish AF from other similar lesions [11].

Differential diagnoses for AF include several odontogenic and nonodontogenic tumors [8, 9]. For example:

- Ameloblastoma: Presents as multilocular radiolucency with a “soap‐bubble” appearance but lacks dental papilla‐like stroma.
- Odontogenic myxoma: Shows a “honeycomb” or “soap‐bubble” radiographic pattern and contains small inactive rests of odontogenic epithelium microscopically.
- Dentigerous cyst: Typically associated with an unerupted tooth and characterized by a unilocular radiolucency surrounding the crown.
- Central ossifying fibroma: Features well‐defined radiolucency with calcifications and often causes cortical expansion.
- Ameloblastic fibrosarcoma: Distinguished by the presence of mitotic cells and atypical mitosis.

Although AF is considered benign, it has a recurrence potential and, in rare instances, can transform into AFS [4]. Approximately 24% of AFS cases arise from benign or recurrent AF. Immunohistochemical markers are instrumental in differentiating AF from AFS; the mesenchymal component of AF is negative for Ki67, PCNA, and P53, whereas AFS shows positive staining for these markers [2, 11].

Treatment for AF typically involves surgical enucleation and curettage to ensure complete removal while preserving surrounding structures. Partial resection may be necessary for extensive or recurrent lesions. In cases involving significant jaw defects postsurgery, reconstruction may be required to restore function and aesthetics. Regular follow‐up is crucial to monitor for recurrence or malignant transformation, ensuring favorable long‐term outcomes [12].

## 6 Patient Perspective

The patient was initially anxious about the jaw swelling but felt reassured after understanding the benign nature of the condition and treatment plan. The patient expressed relief following successful surgery and remains committed to follow‐up care to monitor for recurrence.

## 7 Conclusion

AF is a rare, benign odontogenic tumor that predominantly affects children and adolescents. In this case, the 8‐year‐old patient presented with a painless swelling in the posterior mandible, diagnosed and managed successfully through surgical enucleation and curettage. The lesion showed typical histopathological features, and the patient is currently undergoing regular follow‐up to monitor for recurrence. Early detection and appropriate management are crucial in minimizing complications, ensuring favorable outcomes, and preventing recurrence.

## Author Contributions

**Priyambada Karna:** conceptualization, data curation, formal analysis, visualization, writing – original draft, writing – review and editing. **Varun Rastogi:** conceptualization, data curation, formal analysis, project administration, resources, supervision, validation, visualization, writing – original draft, writing – review and editing. **Dilasha Dhungel:** conceptualization, data curation, formal analysis, visualization, writing – original draft, writing – review and editing. **Sandhya Chaurasia:** conceptualization, data curation, formal analysis, visualization, writing – original draft, writing – review and editing.

## Ethics Statement

The authors have nothing to report.

## Consent

Written informed consent was taken from the patient for their anonymized information to be published in this article.

## Conflicts of Interest

The authors declare no conflicts of interest.

## Data Availability

The authors have nothing to report.
